# Impaired motor inhibition during perceptual inhibition in older, but not younger adults: a psychophysiological study

**DOI:** 10.1038/s41598-024-52269-z

**Published:** 2024-01-23

**Authors:** Rebecca Healey, Megan Goldsworthy, Sauro Salomoni, Simon Weber, Sarah Kemp, Mark R. Hinder, Rebecca J. St George

**Affiliations:** 1 Sensorimotor Neuroscience and Ageing Research Laboratory, School of Psychological Sciences, College of Health and Medicine, University of Tasmania, Hobart, Australia; 2 Integrative Model-Based Cognitive Neuroscience Research Unit, Department of Psychology, University of Amsterdam, Amsterdam, The Netherlands

**Keywords:** Human behaviour, Cognitive ageing, Cognitive control, Motor control

## Abstract

The prefrontal cortex (PFC) governs the ability to rapidly cancel planned movements when no longer appropriate (motor inhibition) and ignore distracting stimuli (perceptual inhibition). It is unclear to what extent these processes interact, and how they are impacted by age. The interplay between perceptual and motor inhibition was investigated using a Flanker Task, a Stop Signal Task and a combined Stop Signal Flanker Task in healthy young (n = 33, Mean = 24 years) and older adults (n = 32, Mean = 71 years). PFC activity was measured with functional near-infrared spectroscopy (fNIRS), while electromyography (EMG) measured muscle activity in the fingers used to respond to the visual cues. Perceptual inhibition (the degree to which incongruent flankers slowed response time to a central cue) and motor inhibition (the speed of cancellation of EMG activation following stop cues) independently declined with age. When both processes were engaged together, PFC activity increased for both age groups, however only older adults exhibited slower motor inhibition. The results indicate that cortical upregulation was sufficient to compensate for the increased task demands in younger but not older adults, suggesting potential resource sharing and neural limitations particularly in older adults.

## Introduction

In the field of cognitive neuroscience, the term “inhibition” implies control over psychological and behavioural processes to adapt to environmental demands. For example, motor inhibition (i.e., the ability to cancel planned movements when they are no longer appropriate) compels us to stop reaching forward when sensing radiant heat from an iron, while perceptual inhibition (i.e., the ability to filter relevant from irrelevant sensory information) allows us to ignore distracting sensory information when driving down a busy street. The prefrontal cortex (PFC) is known to play a key role in inhibitory behaviours^1–3^ but is, however, particularly prone to neural degradation in normal ageing^4^. A recent meta-analysis of extant behavioural data concludes that while motor inhibition declines with age, perceptual inhibition remains relatively stable^5^, suggesting the processes may be controlled via independent mechanisms. However, other work has concluded that because behavioural deficits are observed when perceptual and motor inhibition are performed *together* (compared to alone) they may use common cognitive resources^6–9^. The current study assesses perceptual and motor inhibition using a paradigm that engages each process independently and in combination. Functional imaging of the PFC was considered together with behavioural and physiological data to gain a comprehensive understanding of the neural underpinnings of perceptual and motor inhibition and how they are impacted by ageing.

Older adults experience higher incidence of accidents, injuries and falls, which contribute to significant social and healthcare costs within ageing societies^10^. In daily life, the need to quickly cancel planned motor actions often occurs while perceptual inhibition processes are also engaged. A reduced capacity to simultaneously perform two types of inhibitory control may contribute to adverse outcomes. For example, imagine rushing through a busy train station. You need to use perceptual inhibition to ignore the barrage of irrelevant visual and auditory input to focus on the key information that will guide you to your platform. If the person in front of you suddenly stops walking, you must engage motor inhibition to abruptly stop walking to avoid a collision. Safely navigating this situation is contingent on optimal concurrent perceptual and motor inhibition function.

An understanding of perceptual and motor inhibition processes cannot be achieved by measuring the overt behaviours alone. Functional brain imaging is needed for a more complete understanding. For example, young and older adults show no ostensible difference in behavioural performance on the Flanker task—a paradigm that indexes perceptual inhibition via the response time cost and accuracy decrement of responding to a target arrow flanked by arrows of incongruent compared to congruent directions^5^. However, concurrent neuroimaging during this task shows differences between young and older adults. Both fMRI^11^ and EEG research^12^ show there is increased neural activity in older adults compared to younger adults during incongruent flanker trials. Similar effects have been observed in motor inhibition tasks, with fMRI showing older adults have higher neural activity compared to younger adults—however, a reduction in activity for more difficult tasks is associated with impaired behavioural performance in older adults^13^. These findings may be interpreted within the CRUNCH (Compensation-Related Utilization of Neural Circuits Hypothesis) theory of cognitive ageing, whereby increased cortical activation in older adults supports behavioural task performance by compensating for neuronal loss—however, once task load exceeds resource capacity, performance and brain activity declines^14–16^.

Functional near infrared spectroscopy (fNIRS), is a neuroimaging technique that records haemodynamic changes of cortical tissue to infer neural activity in a similar way to the BOLD response of fMRI^17^. FNIRS has a higher spatial resolution than EEG, although lower temporal resolution due to the haemodynamic response delay. It is relatively robust to motion artefacts so is recommended during freely moving tasks^18–20^.

Action cancellation is a latent process however stopping speed can be estimated using the Stop Signal Task (SST)^21,22^. During the SST, participants respond as quickly as possible to ‘go’ sensory cues but must stop their incipient response on a small proportion of trials when ‘stop’ cues are presented; Stop Signal Reaction Time (SSRT) is estimated from the time delay between go and stop cues when the likelihood of stop success if 50%. However, this estimate of stopping speed cannot distinguish between successful stopping due to motor inhibition, a waiting strategy, or a lapse in attention leading to a delayed response i.e., responses to the go stimulus are never initiated so motor inhibition is not engaged on a successful stop trial. A recently-developed approach uses electromyography (EMG) of effector muscles to identify trials in which movement is initiated (through a partial EMG response in the cued hand), but successfully inhibited a there is no subsequent button press^23–25^. Thus, stopping speed calculated from partial EMG responses, is a veridical measurement of motor inhibition, isolated from waiting strategies and poor attention. In the current study, this technique was used for the first time to compare motor inhibition in older and young adults in a standard choice SST, and to assess how motor inhibition is affected when perceptual inhibition is also engaged.

According to the Multiple Sources Theory^26^, when perceptual and motor inhibition tasks are engaged simultaneously, the observed performance deficit compared to either process assessed in isolation infers the degree of overlap in cognitive resources used by the two processes. The Stop Signal Flanker Task (SSFT) combines the stop-signal (motor inhibition) and flanker (perceptual inhibition) tasks. When stop cues are presented on trials with incongruent flankers, participants are required to engage both perceptual and motor inhibition processes. Prior research suggests that estimated stopping speed (SSRT) is longer for incongruent trials^6–8,27,28^ compared to congruent trials^8,9^ in young adults.

To investigate how age impacts the interaction between motor and perceptual inhibition processes, this study recorded behavioural, physiological and neural responses during the SST, Flanker Task and the combined SSFT. To our knowledge, this is the first study to compare young and older adults’ performance on the SSFT with functional neuroimaging. Previously, three studies have performed functional neuroimaging of the SSFT task in young participants^27–29^ with two of them suggesting involvement of inferior frontal regions of the PFC in both motor and perceptual inhibition^27,28^. Given CRUNCH theories of cognitive control, we hypothesised increased levels of neural activity and/or more widespread activity as the inhibitory demands of the task increase, i.e., we would expect successful stopping on incongruent trials of the SSFT task to have relatively higher levels of neural activity compared to successful stopping on congruent trials of the SSFT, particularly in older adults.

## Results

**Figure 1:**
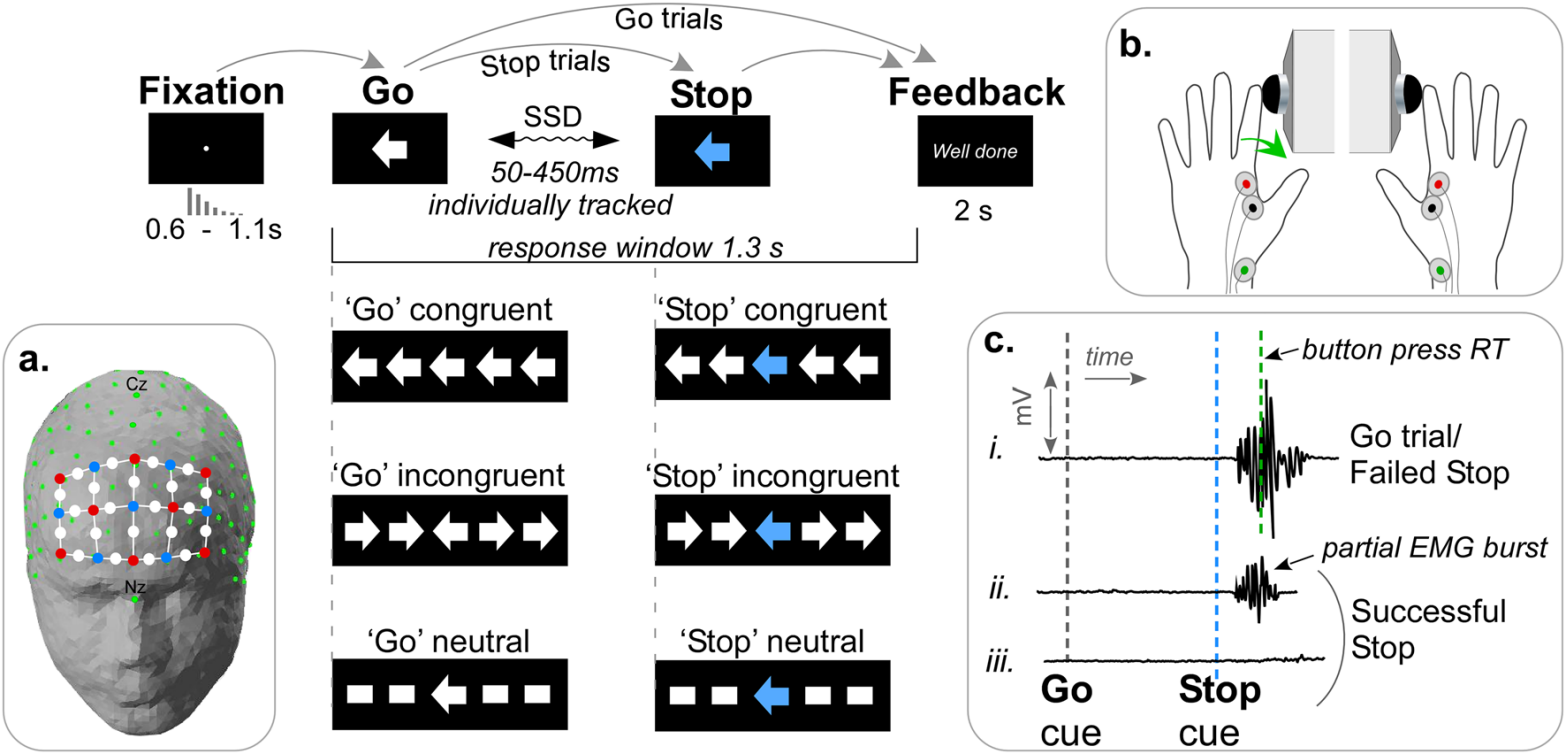
Task stimuli and the methodological approach. the main figure shows the trial sequence for each of the conditions. Participants were comfortably sat approximately 60 cm from a 27” monitor. At the beginning of each trial, a central *Fixation* dot was displayed on the screen. The duration of the fixation varied with a truncated exponential distribution (range: 0.6–1.1 s) to prevent anticipation of the timing of the ‘*Go*’ signal. The *Go* signal cued participants to respond to the direction of the central arrow (left or right) with abduction of the corresponding index finger (inset image **b**). On 30% of the trials of the SST, and SSFT conditions the white arrow changed colour to blue after an individually tracked stop signal delay (SSD). The colour change indicated participants should *Stop* the button press. *Feedback* about trial performance was displayed for 2 s. Depending on trial performance, the feedback for ‘Go’ trials was either: “You’ve slowed down”, “Incorrect”, or “RT = … “. The feedback for ‘Stop’ trials was either: “Correct” or “Incorrect”. Following this feedback, a blank screen was presented for 2 s before the start of the next trial. Example visual stimuli for the CRT (Go trials only) and SST (both Go and Stop trials) conditions are presented in the top row. The lower rows show stimuli for the Flanker Task (Go trials only) and SSFT condition (both Go and Stop trials). Inset image (**a**) shows the montage of fNIRS channel locations (white dots), located midway between fNIRS sources (red dots) and detectors (blue dots) with 30 mm source-detector separation. Neuro-navigation coordinates (10–20 system) are also depicted (green dots). Inset image (**c**) shows representative EMG bursts (**ci**) depicts a typical EMG response when pressing the button—either for a standard Go trial or a failed stop trial. (**cii**) depicts a typical *partial EMG* response, i.e., a muscle response on the side of the cued hand was initiated but the button press was successfully aborted. (**ciii**) depicts a successful stop in which there was no muscle response initiated.

Groups of young and older adults completed four response selection tasks as shown in Fig. 1: Choice Reaction Time (CRT), Stop Signal Task (SST), Flanker Task (FT) and the Stop Signal Flanker Task (SSFT). During each trial presentation, synchronised recordings were made of neural activity across the PFC (Fig. 1a), and effector muscles of each index finger (Fig. 1b,c)—see “Methods” for details.

### Stopping performance

**Figure 2:**
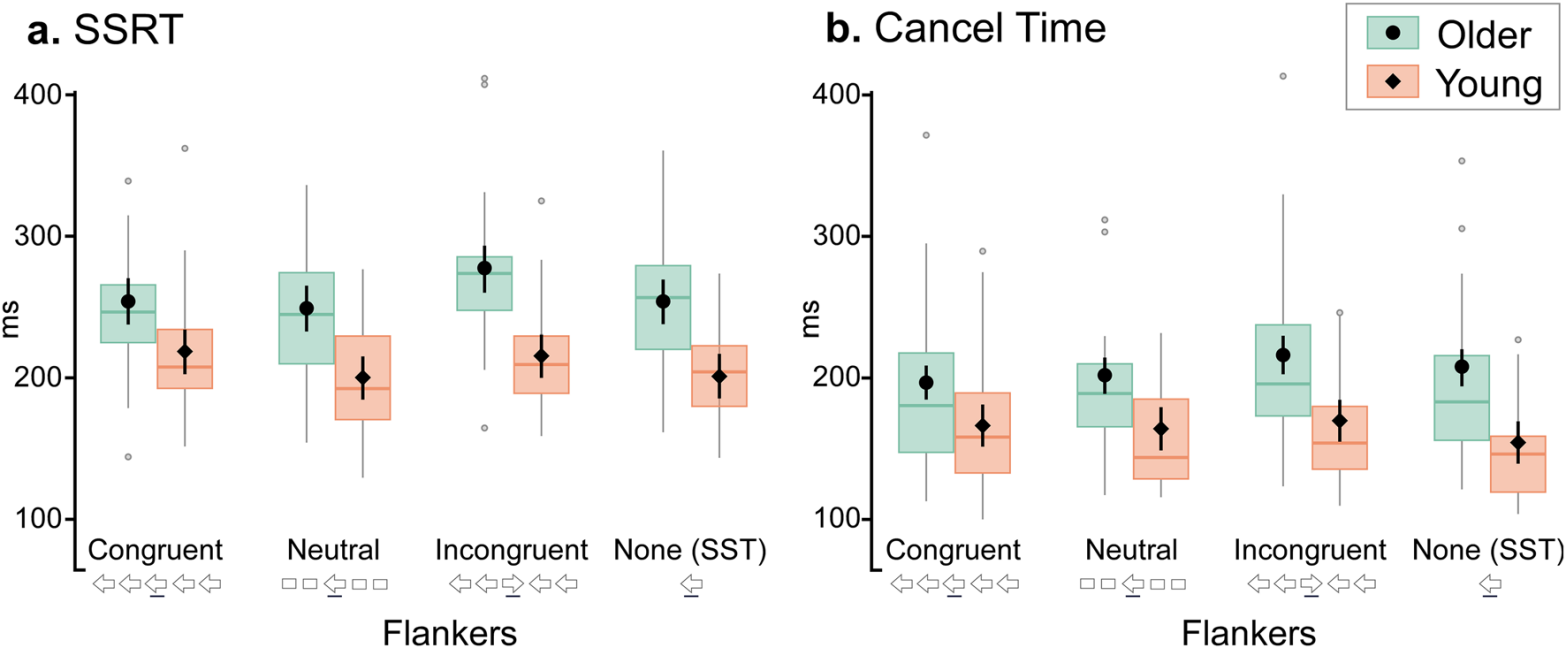
SSRT and Cancel Time measures of stopping speed. Changes in SSRT (**a**) and Cancel Time (**b**), for young and older adults. Black symbols represent group mean for each level of Flanker congruency (older: circles; young: diamonds); with black lines indicating the 95% confidence intervals around the mean. Additionally, boxplots indicate the median, interquartile range, maximum, and minimum of the data. Outliers (points > 1.5 times the interquartile range) are shown as grey points. Note that Cancel Time was faster than SSRT and that regardless of the way motor inhibition was operationalised (SSRT or Cancel Time), older adults demonstrate longer inhibition times for incongruent stop trials relative to other congruency conditions.

The speed at which a response was able to be stopped in the SST and SSFT is presented in Fig. 2, with descriptive data presented in Supplementary Table S1 online. Stopping speed was either estimated from the latency of overt button presses via SSRT (Fig. 2a), or via physiological measures of muscle activation Cancel Time (Fig. 2b).

#### SSRT

The main effect of Age group was significant, *F* (1, 62.95) = 29.25, *p* < 0.001, with slower SSRTs in older than young adults (*M*_diff_ = 49 ms, *SE* = 9.13, *t* = 5.41 *p* = 0.001, 95% CI [31 ms, 68 ms], *d* = 7.59). The main effect of congruency was also significant, *F* (3, 178.03) = 6.15, *p* < 0.001. SSRT was slower when there were incongruent flankers compared to when there were neutral flankers (*M*_diff_ = 22 ms, *SE* = 5.55, *t* = 3.91, *p* = 0.001, 95% CI [7 ms, 37 ms], *d* = 0.46) and compared to no flankers on SST trials (*M*_diff_ = 19 ms, *SE* = 5.55, *t* = 3.36, *p* = 0.006, 95% CI [4 ms, 34 ms], *d* = 0.40). All other comparisons across levels of congruency were not significant as was the interaction between Age group and congruency, *p* > 0.05.

#### Cancel time

**Figure 3:**
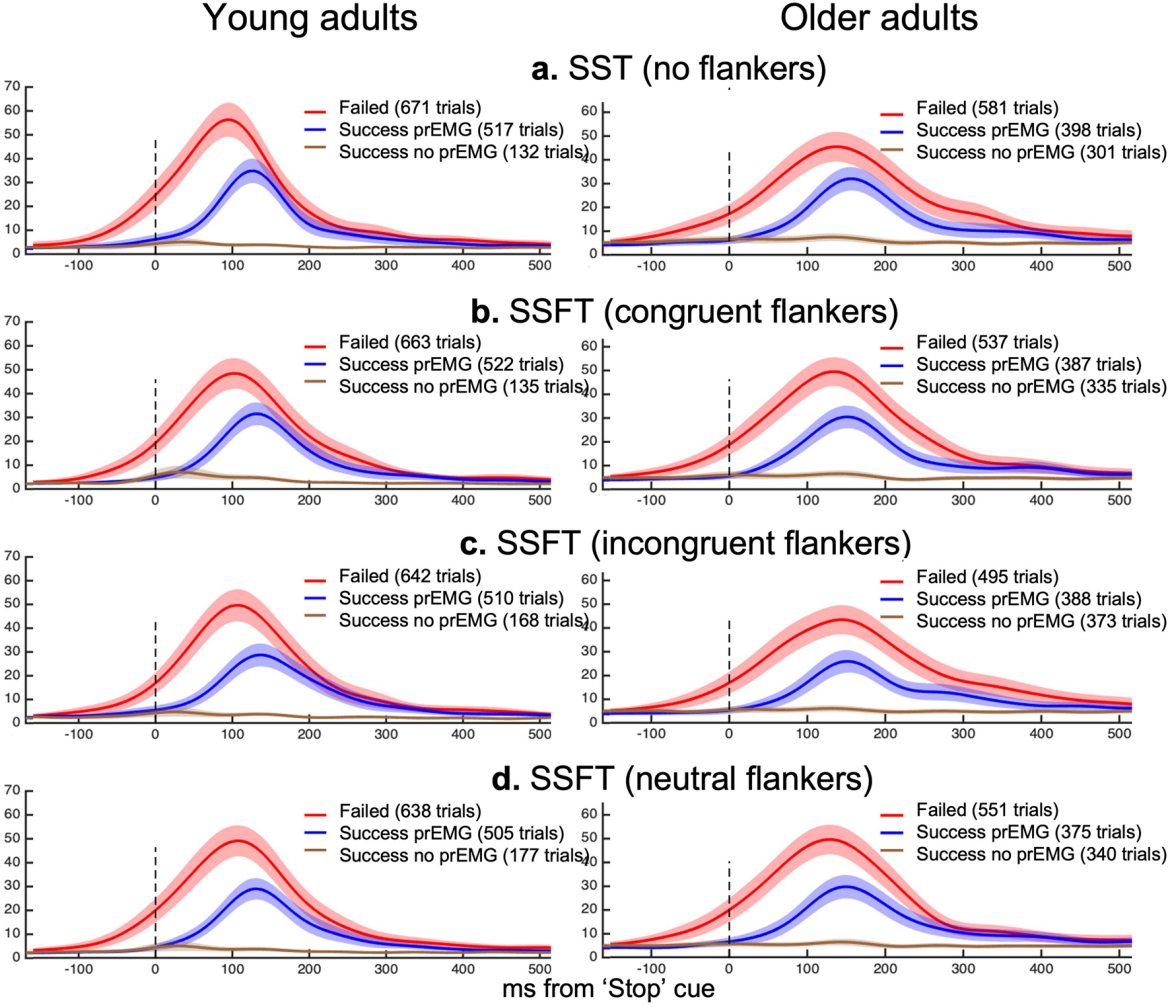
Characteristics of partial EMG bursts in stopping trials. Changes in stopping latency and peak EMG amplitude (both prEMG, and RT-generating EMG bursts) for young adults (left column) and older adults (right column). The x axis shows time relative to the stop signal, and the y axis shows the amplitude of prEMG bursts on unsuccessful and successful stop trials. The y axis shows normalised units relative to the average peak EMG amplitude across successful Go trials from the CRT condition for that participant.

The mean profile of the EMG bursts on stop trials, which were used to calculated Cancel Time, are presented in Fig. 3. There was a significant main effect of Age on the proportion of stop trials with prEMG, *F* (1, Inf) = 13.86, *p* < 0.001; *Young* = 39% of total stop trials (95% CI [35%—42%]), *Older* = 30% of total stop trials (95% CI [27–33%]). There were no other significant main effects or interactions.

There were significant main effects of Age group (*F* [1, Inf] = 27.44, *p* < 0.001) and congruency (*F* [3, Inf] = 6.42, *p* < 0.001) on Cancel Time as well as a significant two-way interaction (*F* [3, Inf] = 9.94, *p* < 0.002) between these factors (see Fig. 2b).

In young adults, Cancel Times in all flanker types in the FSST condition (incongruent, neutral, and congruent flankers) were slower compared to the SST condition in which there were no flankers (*M*_diff Incongruent−SST_ = 15 ms, SE = 3.44, z = 4.25, *p* < 0.001, 95% CI [6 ms, 24 ms], *d* = 0.34; *M*_diff Neutral−SST_ = 10 ms, *SE* = 3.36, *z* = 2.83, *p* = 0.028, 95% CI [1 ms, 18 ms], *d* = 0.21; *M*_diff Congruent−SST_ = 12 ms, *SE* = 3.22, *z* = 3.68, *p* = 0.001, 95% CI[3 ms, 20 ms], *d* = 0.28). No other comparisons between flanker types were significant. In contrast, for older adults, Cancel Times for the incongruent flanker condition were significantly slower than for the congruent flanker condition (*M*_diff Congruent−Incongruent_ = − 20 ms, *SE* = 4.25, *z* = − 4.63, *p* < 0.001, 95% CI [− 31 ms, − 8 ms], *d* = − 0.54) and the neutral condition (*M*_diff Neutral−Incongruent_ = − 14 ms, *SE* = 4.67, *z* = 1.89, *p* = 0.014, 95% CI [− 2 ms, − 27 ms], *d* = 0.39. Additionally, Cancel Time for the SST (no flanker) condition was slower than for the congruent condition (*M*_diff Congruent−SST_ = − 11 ms *SE* = 3.86, *z* = − 2.81*, p* = 0.030, *d* = − 0.31, 95% CI [− 21 ms, − 1 ms]). No other comparisons were significant.

### Performance on go trials

**Figure 4:**
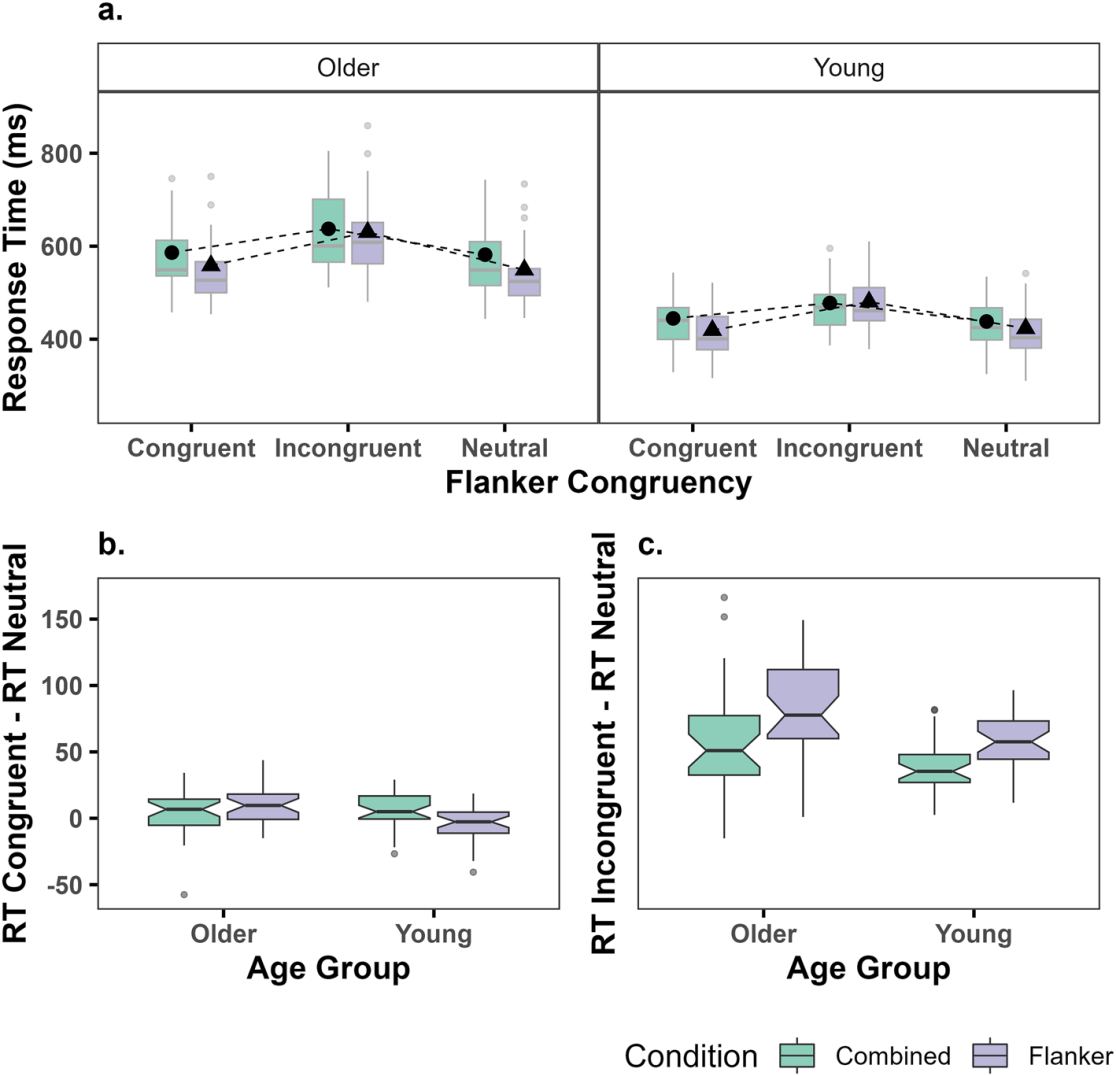
The effects of Age, Congruency and Condition on Reaction Time. Boxplots represent the median and IQR of data stratified by condition (Green = Combined, Purple = Flanker); with whiskers representing the maximum and minimum range values and outlier data (> 1.5 times the IQR) represented by grey dots. In panels B and C, horizontal lines represent median delta RT values, notches represent 95% CIs around the median, and outliers are indicated by black dots. (**a**) RT stratified by Age group, condition and congruency. Black dots represent mean RT for Congruent, Incongruent, and Neutral trials, with black lines representing the 95% CI around the means. (**b**) ΔRT _Congruent−Neutral_ Trials, stratified by Age group and Condition. (**c**) ΔRT _Incongruent−Neutral_ Trials, stratified by Age group and Condition.

The speed at which young and older adults responded to Go trials within the different conditions are presented in Fig. 4 (with descriptive data available in Supplementary Table S2 online). Response time on Go trials was influenced by significant main effects of Age group, (F[1, Inf] = 4595.85, *p* < 0.001), Condition (*F* [1, Inf] = 46.31, *p* < 0.001) and Congruency (*F* [2, Inf] = 1426.64, *p* < 0.001)*.* Go trial RTs were longer in older than young adults; and were longer on incongruent trials relative to all other trial types. However, results are best interpreted in terms of a significant three-way interaction between Age group, Condition (Flanker/SSFT), and congruency (congruent, neutral, incongruent) (*F* [2, Inf] = 63.83, *p* < 0.001).

#### RT to incongruent relative to neutral flankers

In both young and older adults, RT was slower on incongruent compared to Neutral trial types on both the Flanker task (ΔRT _Young_ = 57 ms, *SD* = 11.95, *z* = 27.60, *p* < 0.001, 95% CI [51 ms, 64 ms], *d* = 2.29; ΔRT _Older_ = 81 ms, *SD* = 11.65, *z* = 39.31, *p* < 0.001, 95% CI [75 ms, 87 ms], *d* = 4.51) and the SSFT task (ΔRT _Young_ = 40 ms, *SD* = 9.48, *z* = 23.97, *p* < 0.001, 95% CI [35 ms, 44 ms], *d* = 2.57; ΔRT _Older_ = 56 ms, *SD* = 9.62, *z* = 32.95, *p* < 0.001, 95% CI [51 ms, 61 ms], *d* = 4.23). The magnitude of this effect was greater in older than young adults in both the Flanker condition (ΔRT _Older_ − ΔRT _Young_ = 24 ms, *SD* = 17.58, *z* = 10.85, *p* < 0.001, *d* = 2.00) and in the SSFT condition (ΔRT _Older_ − ΔRT _Young_ = 16 ms, *SD* = 12.90, *z* = 9.99, *p* < 0.001, *d* = 1.68) (see Fig. 4c).

#### RT to congruent relative to neutral flankers

The significant interaction appears to be driven by differences in the way young and older adults responded to trials with congruent flankers (see Fig. 4b). For young adults, there was no significant difference in RT for Congruent and Neutral trials of the Flanker task (ΔRT _Congruent−Neutral_ = − 4 ms, *SD* = 9.48, *z* = − 2.35, *p* = 0.278, 95% CI [− 9 ms, 1 ms], *d* = 0.16); in contrast, congruent trial RTs were longer than neutral trial RTs in older adults (ΔRT _Congruent−Neutral_ = 4 ms, *SD* = 9.33, *z* = 2.35, *p* = 0.278, 95% CI [< 1 ms, 9 ms], *d* = 0.55)*.* For the SSFT, young adults had slightly slower RTs for Congruent than Neutral trials (ΔRT _Congruent−Neutral_ = 7 ms, *SD* = 7.35, *z* = 5.12, *p* = 0.001, 95% CI [3 ms, 10 ms], *d* = 0.46); this was replicated in older adults (ΔRT _Congruent−Neutral_ = 4 ms, *SD* = 6.28, *z* = 4.01, *p* = 0.001, 95% CI [1 ms, 8 ms], *d* = 0.31). There was no significant difference in the magnitude of this effect with age, (ΔRT _Older_ − ΔRT _Young_ = 2 ms, *SD* = 8.47, *z* = − 1.98, *p* = 1.00, *d* = 0.43).

#### Peak EMG amplitude on go trials

**Figure 5:**
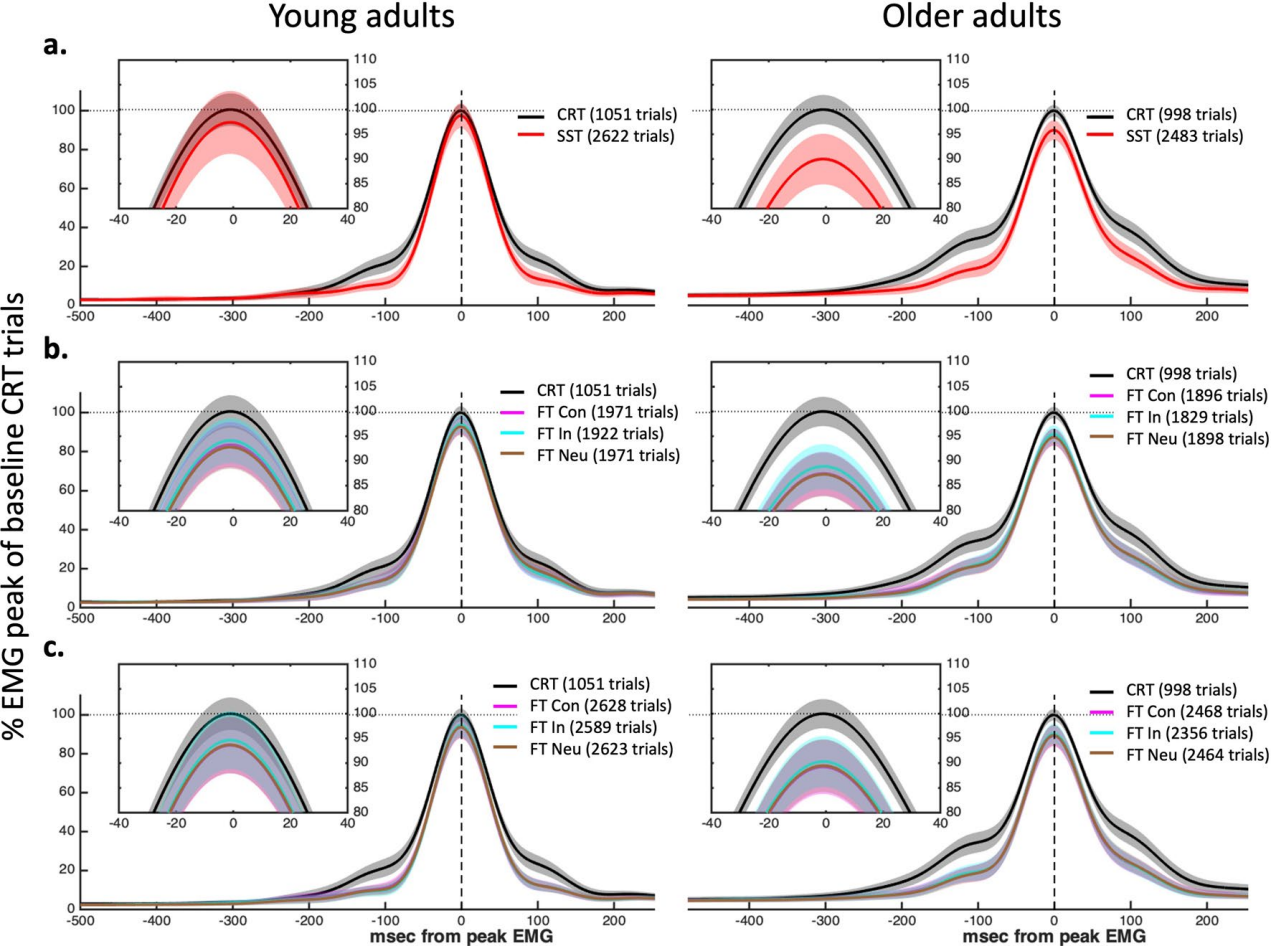
EMG mean response profiles in Go trials for Young (left) and Older participants (right). Each condition (**a**) SST, (**b**) Flanker, and (**c**) SSFT is compared to the CRT condition (grey) by synching peak EMG mean (± 95% CI) waveforms. The inset figures are zoomed in on the peak to highlight comparisons between peak amplitudes. The ordinate axis shows normalised units relative to the average peak EMG amplitude across successful Go trials from the CRT condition for that participant.

The normalised peak EMG amplitude reflects the neural drive initiated to perform the behaviour. Difference in peak amplitude for RT-generating burst are shown in Fig. 5. There was a significant two-way interaction between Age group and Congruency on the peak amplitude of RT-generating EMG bursts, *F* (7, Inf) = 5.50, *p* < 0.001. Specifically, peak EMG amplitude was significantly lower for all trial types compared to CRT in older adults (estimate range = 10.03–12.71%, *p* < 0.001, *d* range = 0.51–0.72) for all trial types. In contrast, in young adults, EMG peak amplitude was significantly lower for all trial types relative to the CRT condition (estimate range = 3.76–7.21%, *p* < 0.001, *d* range = 0.15–0.41) except for SST trials (estimate = 2.58, *z* = 2.96, *p* = 0.085). When young and older adult performance was compared for each trial type (e.g., Congruent _Young_ − Congruent _Older_), no comparisons were significant at the 0.05 level (*d range* = 0.27–0.43 for all estimates), suggesting that while decreased neural drive in more complex decision tasks became more pronounced with age, this is not statistically significant.

Additional analyses comparing the baseline conditions with the experimental conditions are presented in Supplementary Material S3 (exploring the effect of visual complexity on RT) and Supplementary Material S4 online (exploring the extent of proactive slowing of go responses during the SST condition relative to the CRT condition).

### Prefrontal cortical activity

**Figure 6:**
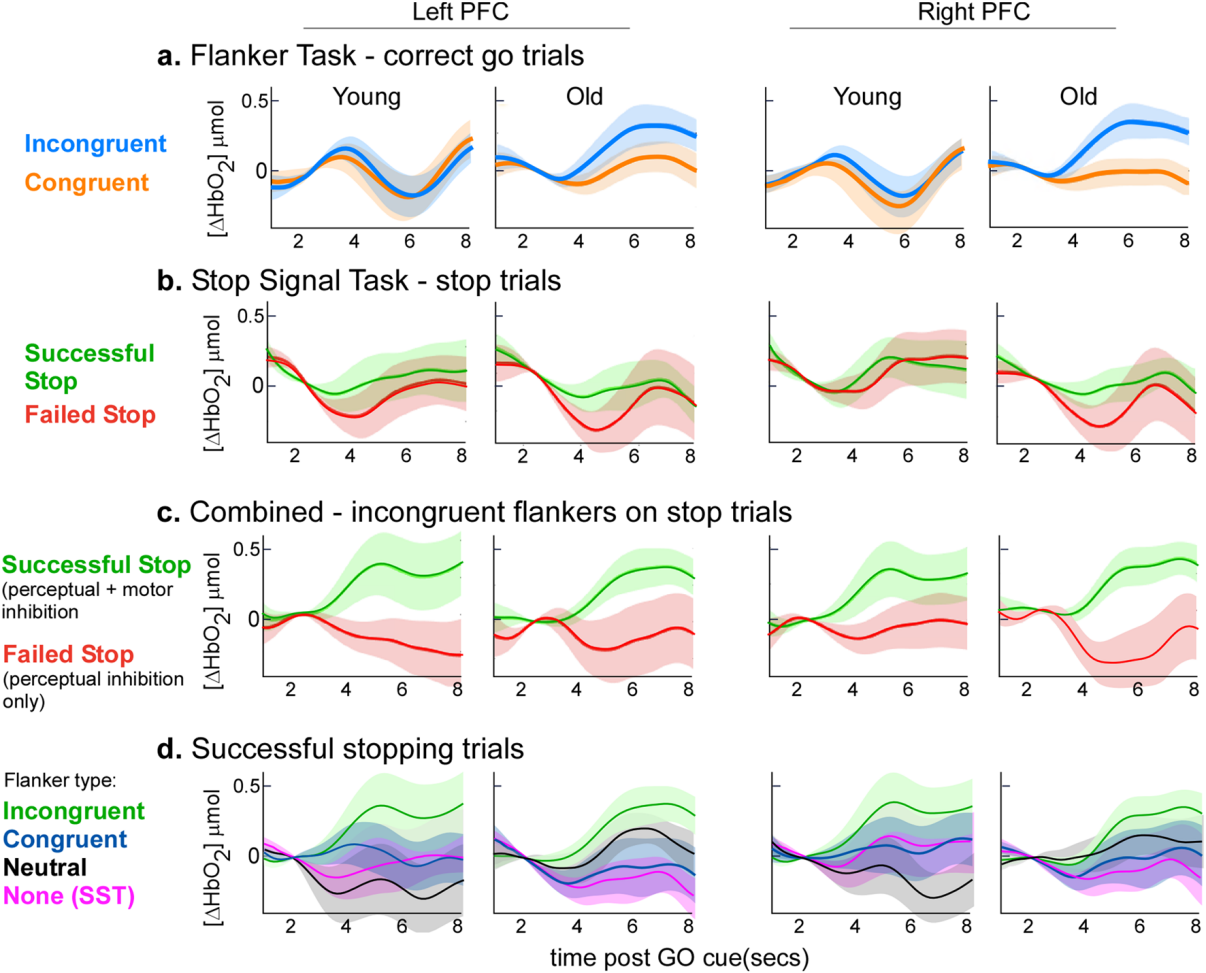
Prefrontal event-related haemodynamics changes. Mean changes (± 95% CI bands) in oxygenated haemoglobin in the left (left panels) and right (right panels) PFC. Activity was aligned to go stimulus onset and zeroed over the mean level 0−2 s. Row (**a**) represents neural activity changes for congruent (orange lines) and incongruent (blue lines) Go trials of the Flanker task. Row (**b**) represents changes in oxygenated haemoglobin for successful and failed stop trials of the SST. Row (**c**) represents oxygenated haemoglobin changes for successful and unsuccessful incongruent stop trials of the combined SSFT task. Row (**d**) shows the PFC activity for successful stop trials by flanker type in the SSFT condition.

**Figure 7:**
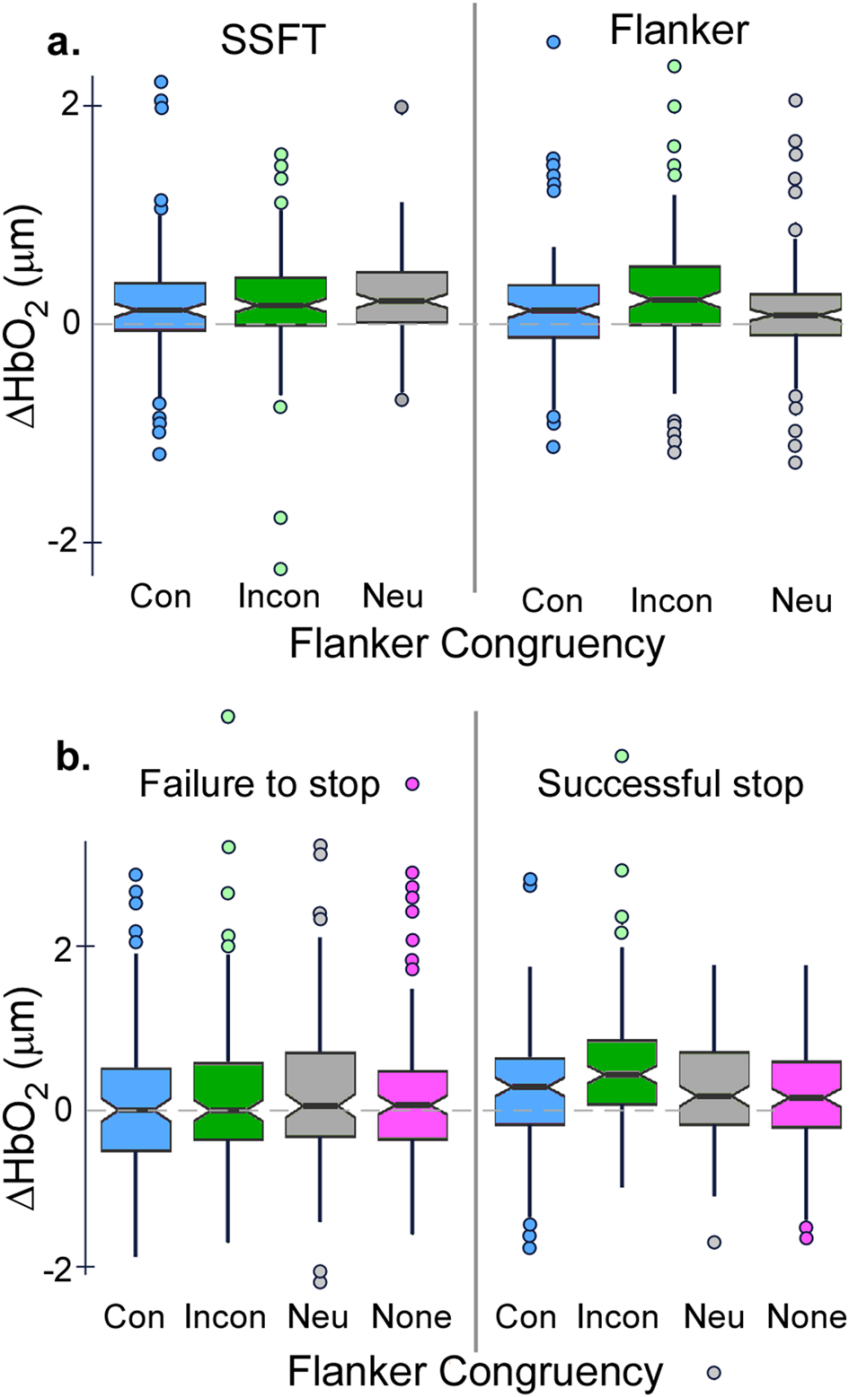
Changes in neural activity. (**a**) Changes in bilateral prefrontal neural activity on Go trials of the Flanker and SSFT tasks. As there was no interaction between Age group and other variables, this panel pools data for young and older participants across both left and right PFC. (**b**) Changes in bilateral prefrontal neural activity on Stop trials for the range of different flankers presentations: None (SST task), and Congruent (Con), Incongruent (Incon) and Neutral (Neu) of the SSFT. For successful stop trials (right panel), bilateral neural activity change was greater for the Incongruent stop trials relative to all other levels of Congruency. Prefrontal neural activity change was greater for incongruent stop-success trials than incongruent stop-fail trials (left panel).

Mean waveforms of the concentration change in oxygenated haemoglobin in the left and right PFC are shown in Fig. 6 with key output parameters presented in Fig. 7.

#### Effect of Flanker congruency on neural activity

During Go trials, there was a significant two-way interaction between Condition (Flanker/FSST) and Congruency, *F* (1, 732) = 4.40, *p* = 0.013. Bonferroni adjusted contrast tests revealed that PFC neural activity increased to a greater extent for incongruent than neutral trials of the Flanker condition, (*M* difference = 0.18, *SE* = 0.05, *t* = 3.54, *p* = 0.006, *d* = 0.46); and was also greater on neutral trials of the combined condition than neutral trials of the Flanker condition (*M* difference = 0.16, *SE* = 0.05, *t* = 0.039, *d* = 0.39). Four- and three-way interactions between Age group, Hemisphere, Condition, and Congruency were non-significant, and no main effects were significant.

#### Effect of stopping on neural activity

There was a significant two-way interaction between Congruency and Stop success, *F* (3, 912.10) = 4.40, *p* = 0.004. Bonferroni-adjusted post-hoc tests revealed that PFC neural activity was significantly lower for Incongruent stop fail than Incongruent stop success trials (*M diff fail* − *success* = − 0.39, *SE* = 0.09, *t* = − 4.41, *p* < 0.001, Cohen’s d = − 0.60). On successful stop trials, neural activity change was greatest for incongruent trials relative to all other levels of flanker congruency (*M* difference congruent − incongruent = − 0.32, *SE* = 0.09, *t* = − 3.62, *p* = 0.009, Cohen’s *d* = − 0.49; *M* difference incongruent – neutral = 0.34, *SE* = 0.09,* t* = 3.82, *p* = 0.004, Cohen’s *d* = 0.52; *M* difference incongruent – SST = 0.38, *SE* = 0.09, *t* = 4.23, *p* = 0.001, Cohen’s *d* = 0.57). No other comparisons, or three- or four-way interactions were significant.

#### Association between prefrontal neural activity and behavioural performance

A regression model assessing the association between the extent to which stopping speed was impacted by perceptual inhibition behaviourally (Δ SSRT Incongruent − Δ SSRT Congruent flankers] and the extent to which prefrontal neural activity increased when it was loaded with both perceptual and motor inhibition tasks compared to neither [ΔHbO_2_ congruent Fail − ΔHbO_2_ Incongruent Success] did not reach statistical significance, *F* (2, 54) = 2.34, *p* = 0.106, *R* = 0.28. Δ SSRT [Incongruent − Congruent] did not account for significant variance in ΔHbO_2_, *F* (1, 54) = 1.80, *p* = 0.186; nor did age, *F* (1, 54) = 1.29, *p* = 0.261.

## Discussion

The current study investigated how ageing affects the interplay between perceptual and motor inhibition by assessing prefrontal cortical activity and behavioural performance when tasks assessing these inhibitory processes were performed in isolation, or concurrently. Consistent with our central hypothesis, when both motor and perceptual inhibition were engaged, PFC activity increased for both age groups, with only older adults exhibiting a deficit in performance. These results suggest either perceptual and motor inhibition rely on shared prefrontal resources, or there are lower thresholds of central cortical processing capacity with age.

Motor inhibition was assessed using an approach that isolates motor stopping ability from attentional processes and waiting strategies^23^, as well as the more traditional SSRT method. By estimating the time at which partial response EMG on successful stop trials reaches peak amplitude (and thereafter begins to decrease), it was possible to estimate the time at which the stop process is engaged relative to the stop cue^23^. Cancel Time calculated with this approach is arguably more accurate than SSRT estimation as it allows action cancellation to be quantified on a trial-by-trial basis, identifying when the motor command was inhibited before an overt behavioural response occurred. Standard estimation methods for SSRT yield only one value per subject (per level of each independent variable), whereas the number of Cancel Time estimates is only limited by the number of successful stop trials in which a motor response was initiated. While the proportion of trials with partial EMG responses was somewhat less in our older, compared to younger, cohort (i.e., 54% of successful stops in older, and 77% of successful stops in younger), a large number of trials per group still yielded partial responses, allowing a robust Cancel Time metric. It also suggests that younger adults may be better able to cancel an action when muscle efferent commands have been initiated.

Consistent with past research, estimates of Cancel Time were ~ 100 ms shorter than SSRTs, and mean Cancel Time in the young group on SST trials (155 ms) was consistent with the range of estimates in recent studies (110 ms–166 ms^23–25,30–32^). Longer Cancel Times were observed for older adults (~ 208 ms) and notably when perceptual inhibition was engaged by incongruent Flankers (~ 170 ms in young; 217 ms in older adults).

Our study provides evidence that perceptual inhibition is more disruptive to the stopping process in older than young adults. Specifically, in older adults, Cancel Time on successful stop trials with incongruent flankers was longer than in Congruent and Neutral congruency conditions. In contrast, for young adults there was no difference in Cancel Time between different types of flankers, rather all flanker trials (congruent, neutral and incongruent) exhibited longer Cancel Times relative to the SST condition in which no flanker stimuli were presented. This suggested Cancel Time was fastest when the visual display was less complex. In other words, older adults showed greater slowing in response to incongruent Flankers, above and beyond the slowing that could conceivably be engendered by a more complex visual display.

For both young and older groups, the increase in PFC activity was highest for successful stopping on incongruent flankers compared to successful stopping on all other flanker types (none, neutral dashes, or congruent arrows), this indicates that the increase in neural activity was due the perceptual inhibition requirement of the task, rather than activity associated with recognition of success or dopaminergic reward mechanisms. The CRUNCH, or scaffolding theory of cognitive ageing^14^ suggests that following a decline in neural structure and function, an increase in neural recruitment in older adults is integral to maintain behavioural performance, and a broader and more bilateral range of regions are recruited for tasks that were previously quite lateralised^33,34^. The extent to which additional neural recruitment is able to compensate for functional and structural changes in the cortex to maintain task performance depends on the task requirements and individual ability. Cognitive resources may ultimately be insufficient when task demands are high (i.e., when engaging perceptual and motor inhibition concurrently), in which case performance declines.

The incongruent stop trials represent the most demanding task condition, as the participant must use perceptual inhibition to ignore the incongruent flanker information whilst cancelling their motor action. Both age groups showed increased prefrontal activity in the Incongruent SSFT condition during successful stop trials relative to failed stop trials, which may reflect an increase in the attentional resources required to perform the task. However, only older adults exhibited impaired behavioural performance when trial-level stop data was analysed (i.e., Cancel Time). Whereas, on the simpler SST task, there was an increase in PFC activity *only* for the older adults relative to baseline CRT but no deficit in performance (see Supplementary Materials S4 and S5). Interpreted together, these results align with the CRUNCH model, whereby compensatory cortical recruitment supports behavioural performance on simple tasks, but a resource ceiling is reached for complex tasks, after which performance declines. These results are consistent with those in our previous work^35^ where, in a dual task requiring both difficult balance tasks and a cognitive verbal task, older adults failed to recruit PFC to a great enough extent to prevent deficits in balance performance.

The correlation between the stopping deficit incurred by concurrent perceptual inhibition, and neural activity changes did not reach statistical significance. This result may not be surprising given CRUNCH and scaffolding theories of cognitive ageing predict the relationship between neural activity and performance is non-linear and mediated by cognitive resource capacity, and how difficult an individual finds the particular task—parameters that were not controlled for in the current study.

Previous research has also reported impaired stopping on incongruent stop trials^6,8,9,27,28,36^. According to Multiple Resources Theory^26^, this deficit in performance when the tasks are performed together indicates that perceptual and motor inhibition are controlled by common processing resources. However, central capacity theories^37^ would argue the deficit could be explained by a bottleneck in central processing capacity. Substituting a memory task (for example) in lieu the flanker task in future studies would resolve this.

The “Motor and Perceptual Inhibition Test” (MAPIT)^38^, an alternative task purported to investigate perceptual and motor inhibition, shows no evidence for decreased performance in instances where both types of inhibition are required, suggesting these processes are independent^39^. The divergence from our findings is likely due to the way each inhibitory process has been operationalised. In the SSFT, motor inhibition is the ability to stop initiated actions during the SST, while perceptual inhibition is the ability to filter relevant, from misleading visual information during the Flanker task. MAPIT assesses two variants of the Simon task designed to elicit stimulus-related conflict (involving ignoring arrow location in favour of direction) and response-related conflict (involving responding to directional arrows with the non-cued hand). Resolving these conflicts are referred to as “perceptual -” and “motor inhibition” respectively, but it has been argued that the perceptual component may not engender sufficient cognitive load to elicit true perceptual inhibition^40^. Furthermore, “motor inhibition” as assessed by MAPIT requires response initiation but does not involve any form of action cancellation or stopping.

During incongruent stop trials of the SSFT task, perceptual inhibition was necessarily initiated *prior* to motor inhibition, as response selection (inhibiting the incongruent flankers to select the responding hand-based target stimulus) occurs prior to motor initiation, and subsequent cancellation. In these trials, it was motor inhibition that was impacted by the perceptual inhibition process—perceptual inhibition itself was not affected by the possibility of having to subsequently stop. That is, despite the purported sharing of similar neural resources, it appears that only if motor inhibition is actively engaged (when a stop signal appears) does the PFC activity increase; the mere possibility of stopping does not affect ongoing perceptual inhibitory processes. This finding aligns with a previous study suggesting that during sequential cognitive control tasks, the second task tends to show performance decline as a result of resource sharing^41^. Adjusting the sequential timing of the inhibitory processes in future experimental paradigms may help determine whether there is bidirectional interference between the perceptual and motor inhibition processes and rule out the contribution of order effects.

A significant problem in interpreting the results of motor inhibition research has been participants’ use of slowing strategies^42,43^ in an attempt to facilitate accurate stopping^42^. Although the onset of the stop signal changes iteratively with every trial, these adjustments tend to be relatively small (e.g., 50 ms) and may take a long time to “catch up” with participants who slow down drastically during the task. Moreover, significant slowing in Go trials challenges the assumptions of the horse-race model, whereby *contextual independence* requires that Go trial performance should be invariant regardless of whether or not stop trials may be encountered. In order to mitigate the use of waiting strategies, we used visual feedback throughout the experiment if participants RT became too slow. Prior research has demonstrated that the use of such feedback results in little or no proactive slowing and yields ~ 40% of successful stop trials with prEMG^25^. In the current study, the slowing was limited to < 25 ms across age groups (see Supplementary Materials).

Between-subject dispersion of Cancel Time estimates was more pronounced in the older than the young adult group. This may reflect variation in age-related sensorimotor decline as all older participants scored well on the cognitive screening questionnaire. These differences become somewhat obscured when a central tendency measure (i.e., mean) SSRT is used to operationalise stopping ability. Future research in this field should consider prEMG to measure stopping performance as our research indicates that it is a more powerful and nuanced approach, that is suited to testing inhibitory control within cohorts that vary in terms of their brain health and motor skill. If prEMG can be evoked reliably, then fewer trials are needed, which is an important consideration for measurement of motor inhibition in clinical populations.

In summary, this study used a combination of innovative and established protocols to investigate the interplay between perceptual and motor inhibition at both the behavioural and neural level. The findings suggest that for older adults, motor inhibition is impaired when perceptual inhibition processes are engaged. This deficit may contribute to higher rates or accidents and falls in older adults, particularly in more challenging environments.

## Methods

### Participants

The sample comprised 65 young and older adult volunteers from the Hobart community. The older group (n = 32; 17 female) had a mean age of 71 years (range: 60–90), while the young group (n = 33; 22 female) had a mean age of 24 years (range: 18–36 years). The research was approved by the University of Tasmania Human Research Ethics Committee (Ref #14865) and all participants provided signed, informed consent prior to participation in agreement with the Declaration of Helsinki. Participants were excluded if they had a neurological disorder, prior brain surgery or a metal implant in their skull. The global cognitive status of the older participants was assessed with the standardised Mini-Mental State Examination (sMMSE)^44^. All participants scored above 25 (range 27–30), indicating normal cognitive status.

### Behavioural tasks

Participants were seated comfortably in front of a computer monitor upon which visual stimuli were presented. They completed four different tasks, all requiring motor responses using the left or right index finger by pressing custom-built response buttons mounted in the vertical plane (index finger abduction see Fig. 1b). The first two conditions (Choice Reaction Time task and Flanker task) were presented in a fixed order to establish baseline reaction time (RT) in the absence of any stopping expectation; whereas the order of the subsequent two conditions (Stop Signal Task and Stop Signal Flanker Task) was counterbalanced between participants. Trial types are depicted in Fig. 1, and trial numbers in Table 1. To prevent fatigue, Conditions 3 and 4 were broken into blocks to allow rest breaks.

**Table 1:** Trial numbers for each condition.

Condition	n blocks	Trials/block	Total trials
1. CRT	1	32	32 (+ 8 practice)
2. Flanker	2	90	180 (+ 12 practice)
3. SST	2	60	120 (+ 8 practice)
4. SSFT	4	90	360 (+ 16 practice)

Left and right stimuli were presented with equal frequency. In Flanker and SSFT conditions, trials with congruent, incongruent and neutral flankers occurred with equal frequency. Stop trials occurred on 30% of all SST and SSFT task trials and were equally frequent across all levels of hand and congruency.

#### Condition 1

Choice Reaction Time (CRT): Participants were required to respond as quickly as possible to a centrally-presented arrow with a button press using their index finger of the hand corresponding to arrow direction (left or right).

#### Condition 2

The Flanker task was used to ascertain choice RT when the central arrow was flanked by either congruent or incongruent directional arrows, or by white dashes (neutral stimulus). Slower responses to the incongruent arrows relative to the neutral or congruent arrows indicates worse perceptual inhibition ability. The inclusion of neutral flankers allowed perceptual facilitation and perceptual inhibition to be assessed relative to a condition with similar visual complexity (i.e., five stimuli on the screen, with flankers that don’t provide any faciliatory of inhibitory information in contrast to the single central arrow in the CRT task; see Supplementary Material S3 online).

#### Condition 3

The Stop Signal Task (SST) was used to assess motor inhibition ability. As in the CRT (Condition 1), participants responded as quickly as possible to the direction of a central arrow (i.e., the ‘go’ signal). On 30% of trials, the arrow changed colour (i.e., the ‘stop’ signal, see Fig. 1), indicating that participants should attempt to cancel the button press. The delay between the ‘go’ and ‘stop’ signals (stop signal delay; SSD) was initially set at 200 ms and adjusted in 50 ms increments using an active staircasing procedure, independently calculated for each hand. The SSD increased by 50 ms after successful stop trials, (making stopping on the subsequent trial less likely) and decreased by 50 ms after failed stop trials (making stopping on the subsequent trials more likely). Thus over all stopping trials stop success approached 50% for each hand. Given that go response slowing (i.e., a waiting strategy) undermines the assumptions of the calculations for Stop Signal Reaction Time (SSRT)^21^, feedback (“You’ve slowed down!”) was provided after each trial when RT was > 150 ms above each participant’s mean CRT (calculated from Condition 1). Go responses during SST occurred, on average, less than 25 ms later than during CRT for both young and older adults, suggesting compliance with task instructions (See Supplementary Materials for descriptive statistics).

#### Condition 4

The combined Stop Signal Flanker Task (SSFT) measured the interaction between perceptual facilitation and inhibition, and motor inhibition. Visual stimuli were the same as those used for the Flanker task. However, on 30% of all trials, the central arrow changed colour (as described in the SST condition), indicating that participants should cancel the button press. As per the SST, independent staircasing procedures adjusted SSD for each hand and level of congruency independently to achieve a stop success rate of ~ 50%; in each congruency condition (congruent, incongruent, neutral). Feedback (“You’ve slowed down!”) was provided when go response RTs were > 150 ms slower than that participant’s mean CRT for the comparable condition, i.e., congruent/incongruent/neutral trials from the Flanker task alone (*Condition 2*).

### Physiological measures

#### Electromyography

Cutaneous electromyography (EMG) was recorded using adhesive electrodes (Ag/AgCl) positioned in a belly-tendon montage over the left and right first dorsal interossei (FDI); an additional electrode positioned on the ulnar bone on each wrist was used as ground reference (Fig. 1b). The analogue signals were amplified 1000 times, sampled at 2000 Hz and band-pass filtered between 20 and 1000 Hz, (CED Power 1401 and CED 1902, Cambridge, UK) and saved for post processing.

#### Functional near infrared spectroscopy

The haemodynamic response was recorded with an 8-source, 7 detector fNIRS montage over the medial and dorsolateral prefrontal cortex. The montage of optodes did not correspond directly to EEG coordinates, although the overlap with the 10–20 layout is presented in Fig. 1a. This system (NirSport, NIRs Medizintechnik GmbH, Berlin), designed to be measure PFC activity, uses near-infrared light (emitted at 760 nm and 850 nm wavelengths to capture haemodynamic changes within the outer ~ 1.5 cm of cerebral cortex. Data were recorded with NirStar software (version 15.3) with a sampling rate = 7.8125 Hz.

The experimental protocol was developed in PsychoPy3^45^ software. Synchronisation between the behavioural tasks and the physiological measures was achieved with a digital TTL signal from PsychoPy that triggered EMG data collection for each trial, and also marked a digital event in the continuously collected fNIRS data. The mean delay time between the TTL pulse and the increase in luminosity intensity on the screen (from black, to the white arrow measured via an Arduino photodiode) was 2.9 ms (SD = 2.0 ms), which was less than the refresh rate of the 240 Hz monitor (1frame = 4.2 ms).

### Data processing

Data were analysed in MATLAB (R2017b) and R (R Core Team, 2022) using the packages tidyverse^46^, lme4^47^, emmeans^48^, matrix^49^, lmertest^50^, and multcomp^51^.

#### Stop signal reaction time calculation

SSRT was calculated using the integration method, with go omissions replaced with the subjects’ slowest RT^21^. SSRTs were averaged across left and right hands for each participant by trial type (SST, SSFT-Congruent, SSFT-Incongruent, and SSFT-Neutral). SSRT was only calculated for participants/conditions when assumptions of the “race model” were met, i.e., (i) mean failed stop RTs < mean Go RTs; and (ii) stopping success was between 25 and 75%^21^. As such, four participants’ data were removed for the congruent condition, four for the incongruent condition, two for the neutral condition, and three for the SST condition.

#### Electromyography processing

EMG signals were digitally filtered using a fourth-order band-pass Butterworth filter between 20 and 500 Hz. Onsets and offsets of task-related EMG bursts were detected using a single-threshold algorithm when EMG amplitude was 3 SD above baseline (defined as the lowest activity detected during that trial)^52^. For robustness, EMG bursts separated by less than 20 ms were merged to represent a single burst. Using all defined onsets and offsets within a trial, we defined the RT-generating EMG bursts as the last burst where the onset occurred (i) after the go signal and (ii) at least 50 ms before the button press. Partial EMG responses (prEMG) on stop trials were defined as responses that were initiated after the go stimuli but cancelled (after presentation of the stop signal) and before generating a button press. Specifically, prEMG was defined as (i) EMG onset after the go signal; (ii) time of peak EMG happened after SSD (i.e., inhibition happened in response to the stop signal); (iii) peak prEMG amplitude was greater than 10% of the average peak RT EMG from successful Go trials. EMG signals were full-wave rectified and low-pass filtered at 10 Hz to obtain the EMG profiles. To allow comparisons between conditions, EMG profiles were normalised to the average peak EMG amplitude across successful Go trials from the CRT condition for each participant (Fig. 3). ‘Cancel Time’ was calculated for each prEMG trial as the time from SSD to peak prEMG amplitude^23,24^.

#### fNIRS processing

fNIRS data were processed using established algorithms in Homer3 v 1.80.2 in Matlab (see: https://github.com/BUNPC/Homer3/wiki)*.* Channels with excessively noisy light intensities were removed using the function *Prune Channels* (dRange [1, 3], SNRthresh = 2, SDrange [0, 45]), before being converted to optical density. Regions of motion artifact were then identified with set thresholds that were verified via visual inspection and a principal components analysis was performed only on these segments to avoid over correction in the data (HOMER 3 function: hmrMotionCorrectPCArecurse; tMotion = 0.5, tMask = 1, STDEVthresh = 9, AMPthresh = 100, nSV = 0.97, maximum iterations = 5). The number of components removed varied for each participant to ensure up to 97% of the variance in the segment of data was removed^53^. The fNIRS signals were reconstructed with the remaining components and offset corrected. Data were then low-pass filtered = 0.5 Hz) and converted to concentrations (µmol) of oxygenated haemoglobin (HbO_2_), deoxygenated haemoglobin (HbR) and total haemoglobin (HbT). In line with our prior work, HbO_2_ had the highest signal to noise ratio and was negatively correlated with HbR, so was used as an estimate of *neural activity* for subsequent analyses^20,35^.

fNIRS channels were averaged within left and right hemispheres of the PFC (10 channels on each side excluding the midline channels—see Fig. 1a). Trial-level, waveforms were extracted over a 10-s time window and phase-locked to stimulus onset. Trials were grouped according to Condition type and trial outcomes (success/failure on stopping trials and correct/incorrect response selection on Go trials) and averaged. By averaging over multiple trials, extra-neuronal contributions associated with respiration, pulse rate, Meyer waves etc. were minimised. Cortical activity measured via fNIRS follows a characteristic haemodynamic response function based on the principles of neurovascular coupling; there is an initial dip in the first 1–2 s, followed by a peak and then a return to baseline. The size of the oxygenated haemoglobin change 4–6 s after a neural event (relative to t = 0 s) reflects neural activity associated with that specific event. To account for between subject variability in decision times, the peak change in HBO_2_ concentration within a time window between 4 and 7 s after the stimulus presentation was calculated and used as the measure of neural activity in the statistical analyses.

### Statistical analysis

#### Stopping performance

The effects of Age (young/older), Congruency (SST/neutral/congruent/incongruent) and their interaction effects on the motor inhibition ability measures (SSRT and Cancel Time) were investigated with two mixed models, with a subject-level random effect. Bonferroni-adjusted contrast tests were used to compare changes in stopping performance across all levels of congruency, within young and older adults for significant interactions. For SSRT, a linear mixed model was used, but due to positively skewed, trial-level data for Cancel Time, a generalised model (with a gamma distribution and identity link function) was used. To examine differences in the proportion of trials with and without prEMG, a binomial GLMM was run with a probit link function; with fixed factors of Age and Congruency, and random intercepts for subjects.

#### Go performance

Reaction time data to ‘Go’ trials were screened to remove trials where an incorrect left/right choice response was made and anticipatory responses (RT < 150 ms) (n trials included = 26,639, n trials excluded = 893) as well as practice trials*.* Go RTs were analysed using a generalised linear mixed model (GLMM) with gamma distribution and identity link function. This is consistent with best-practice guidelines for RT analysis, as distributions are typically positively skewed^54^. Fixed effects were Condition (Flanker task; Combined FSST), Congruency (Congruent, Incongruent, Neutral), and Age (young/older), with a subject-level random effect and random slopes by condition. Bonferroni-adjusted contrast tests were used to examine the change in RT for congruent and incongruent flanker trials relative to the neutral flankers. To examine differences in normalised peak EMG amplitude on Go trials between Age group and Congruency | Condition (CRT, SST, Flanker Congruent, Flanker Incongruent, Flanker Neutral, FSST Congruent, FSST Incongruent, FSST Neutral) were analysed with a linear mixed model with subject-level random effect.

#### Neural activity changes

A linear mixed model investigated the effect of flanker congruency on prefrontal neural activity during Go trials. Age group, Hemisphere (left/right), Condition (Flanker/Combined) and Congruency (congruent, neutral, incongruent) were fixed effects, with participant as a random effect. To investigate the effect of stopping on neural activity, linear mixed models examined the effects of Age group, Condition, Hemisphere, and Stop Success (success/fail) on neural activity changes during Stop trial of the SST and FSST conditions with a subject-level random effect.

A regression model was run assessing the effect of Δ SSRT [Incongruent − Congruent] on prefrontal HbO_2_ [Congruent Fail − Incongruent Success]; this allowed us to compare the behavioural effect of perceptual inhibition on motor inhibition, with the corresponding changes in prefrontal activity induced by task demands. Age was included as a factor, with Δ SSRT [Incongruent − Congruent] as a covariate; mean estimates of prefrontal HbO_2_ were merged across both hemispheres.

### Supplementary Information

Supplementary Information.

## Data Availability

The data for this manuscript are not publicly available but may be accessed upon request to the corresponding author.
