# Supplementary material for: Impaired motor inhibition during perceptual inhibition in older, but not younger adults: a psychophysiological study
Source: Sci Rep. 2024 Jan 23;14:2023. doi: 10.1038/s41598-024-52269-z (PMC10805883; doi:10.1038/s41598-024-52269-z)
Supplement: Supplementary file 1 — Supplementary Information. [file 41598_2024_52269_MOESM1_ESM.docx]

**Supplementary Materials**

**Supplementary Table S1**

**Age-Related Changes in Stopping Performance (for SSRT and Cancel Time)**

| ***SSRT*** | | | | ***Cancel Time*** | | |  |
| --- | --- | --- | --- | --- | --- | --- | --- |
| Congruency | *M(SD)** | 95% CI** | p  (stop \| success) *** | *M(SD)** | 95% CI** | *N* obs. P **** | Partial %***** |
| ***Young Adults*** | | | | | | |  |
| Congruent | 253 (52) | 234-273 | 61% | 186 (111) | 175-197 | 4-21 | 79% |
| Incongruent | 277 (59) | 255-301 | 61% | 211 (122) | 199-224 | 3-21 | 75% |
| Neutral | 248 (52) | 229-267 | 57% | 193 (116) | 181-205 | 4-22 | 74% |
| SST | 253 (44) | 237-270 | 55% | 191 (99) | 182-201 | 2-21 | 80% |
| ***Older Adults*** | | | | | | |  |
| Congruent | 219 (44) | 203-229 | 50% | 162 (88) | 154-169 | 6-21 | 54% |
| Incongruent | 216 (39) | 202-229 | 51% | 162 (65) | 157-168 | 8-21 | 51% |
| Neutral | 200 (39) | 186-214 | 52% | 158 (66) | 152-164 | 5-21 | 53% |
| SST | 202 (30) | 192-213 | 49% | 146 (61) | 141-152 | 9-22 | 58% |

* *M*(SD) = Mean (standard deviation) in milliseconds estimates of SSRT and Cancel Time

** 95% CI [lower limit, upper limit] in milliseconds

*** p (stop | success) = probability of successful stop

**** = *N* obs. Pp = Range [lower limit, upper limit] of successful stop trials with prEMG. Lower and upper values indicate the lowest and highest number of prEMG trials observed for *any* participant within each level of Age | Congruency.

***** = Proportion of trials with prEMG (as a percentage of successful stop trials).

**Supplementary Table S2**

**Descriptive statistics for the Three-Way Interaction between Age, Condition, and Congruency on Go Trial Response Time**

Go Trial Reaction Times in Young and Older Adults.

| **Young Adults** | | | | | |
| --- | --- | --- | --- | --- | --- |
| Condition | Arrow Congruency | *M (ms)* | SD *(ms)* | 95% CI (ms) [Lower, Upper] | |
| Flanker | Congruent | 411 | 89 | 408 | 415 |
|  | Incongruent | 471 | 101 | 467 | 475 |
|  | Neutral | 415 | 88 | 411 | 420 |
| Combined | Congruent | 438 | 107 | 434 | 442 |
|  | Incongruent | 469 | 105 | 465 | 473 |
|  | Neutral | 431 | 105 | 427 | 435 |
| **Older Adults** | | | | | |
| Flanker | Congruent | 545 | 123 | 540 | 551 |
|  | Incongruent | 619 | 144 | 612 | 625 |
|  | Neutral | 536 | 113 | 531 | 541 |
| Combined | Congruent | 574 | 138 | 569 | 580 |
|  | Incongruent | 624 | 149 | 618 | 630 |
|  | Neutral | 570 | 138 | 564 | 575 |

**Supplementary Material S3**

**The Effect of Visual Complexity on Reaction Time**

RT slowing was not observed from CRT to neutral Flanker trials; suggesting that RT changes from CRT to congruent and incongruent Flanker trials reflect a pure measure of perceptual facilitation and perceptual inhibition that is not confounded by stimulus complexity.

A generalised mixed model was conducted on data from the CRT block and neutral Flanker trials with a fixed factor of condition, an age by condition interaction, and random slopes Condition | Participant. A gamma distribution was used with an identity link function. Descriptive statistics are reported in Supplementary Table 1.

Results showed no main effect of condition, *F* (1, Inf) = .13, *p* = .721, but a significant Age by Condition interaction, *F* (2, Inf) = 455.99, *p* <.001. Two planned linear contrast tests were used to examine the change in RT from CRT to neutral Flanker trials in young and older adults. In young adults, RTs increased from CRT to neutral Flanker trials by 14ms, *SE* = 4.06ms, *t* = 3.44, *p* <.001; indicating an effect of visual complexity on RT that was not replicated in older adults; equivalent mean difference = -8ms, *SE* = 4.15ms, *t* = 1.90, *p* = .058.

*Supplementary Table S2.*

Descriptive Statistics for the Age * Condition Interaction

| Age | Condition | Mean (ms) | SE (ms) | 95% CI (ms)  [Upper, Lower] | |
| --- | --- | --- | --- | --- | --- |
| Older | CRT | 556 | 5.14 | 546 | 567 |
|  | Neutral Flanker | 547 | 6.18 | 535 | 559 |
| Younger | CRT | 413 | 7.53 | 398 | 428 |
|  | Neutral Flanker | 426 | 7.70 | 411 | 441 |

**Supplementary Materials S4**

**Proactive Slowing – Behavioural Data**

Data from CRT and SST blocks were analysed using a generalised mixed model with a gamma distribution and identity link function. Age group, condition, and the interaction term were set as fixed factors. Random slopes were modelled by subject and condition.

Means and standard deviations are reported in Table 4. There was a significant main effect of Age on RT, *F* (1, Inf) = 1068.13, *p* <.001; and a significant main effect of Condition on RT, *F* (1, Inf) = 8.38, *p* = .004. However, main effects are best interpreted by a significant interaction between Age and Condition, *F* (1, Inf) = 8.67, *p* = .003. Bonferroni-adjusted post-hoc tests indicated that RTs were significant longer in older than young adults in the CRT condition, *estimated difference* = 144ms, *SE* = 3.75ms, *z* = 38.32, *p* <.001; and in the SST condition, *estimated difference* = 132ms, *SE* = 5.41ms, *z* = 25.00, *p* <.001. Younger adult RTs increased from the CRT to the SST condition, *estimate* = 21ms, *SE* = 6.53ms, *z* = 3.18, *p* = .009; but the increase in older adults was smaller, and not statistically significant, *estimate* = 9ms, *SE* = 4.35ms, *z* = 2.11, *p* = .205. These results suggest that proactive slowing from the CRT to the SST condition was observed in younger but not older adults.

*Supplementary Table S3.*

Proactive Inhibition Analysis

Marginal Means and Standard Error for the Age * Condition Interaction

| Age | Condition | Mean (ms) | SE (ms) | 95% CI  [ Upper, Lower] ms | |
| --- | --- | --- | --- | --- | --- |
| Older | CRT | 556 | 3.51 | 549 | 562 |
|  | SST | 565 | 5.81 | 553 | 576 |
| Younger | CRT | 412 | 5.03 | 402 | 422 |
|  | SST | 433 | 8.28 | 417 | 449 |

**Supplementary Materials 5S**

**Proactive Slowing – Functional Neural Imaging Data**

*Statistical analysis*

The effect of proactive inhibition on prefrontal changes in neural activity was explored with a linear mixed model. Only Go trials from the CRT condition (i.e., where stop expectation was absent) and the SST condition (i.e., where stop expectation was present) were included in the model. Age group, Condition (CRT, SST), and Hemisphere were used as fixed effects, and random intercepts were included for subjects.

*Results*

There was a significant main effect of age on prefrontal HbO, *F* (1, 246) = 11.89, *p* <.001, with greater HbO change in older than young adults (*M* Older = .36, *SD* = .17; *M* young = .12, *SD* = .18, Cohen’s *d* = .86). No other main effects or interactions were significant, indicating that engaging proactive inhibition processes did not change prefrontal HbO.


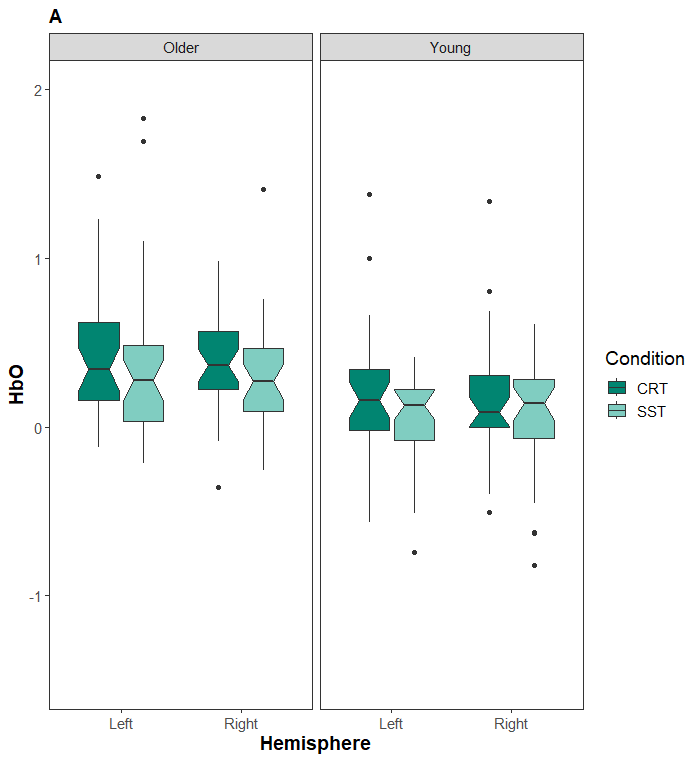


**Supplementary Figure 1. Effect of Stop Expectation on Prefrontal Cortical Activity**

Boxplots represent changes in HbO concentration (micromol) on Go trials by Condition (CRT trials = Dark green; SST trials = Pale green), stratified by age.
